# Research Progress and Prospects of Modified Biochar in the Adsorption and Degradation of Sulfonamide Antibiotics

**DOI:** 10.3390/antibiotics15030268

**Published:** 2026-03-04

**Authors:** Junjie Wang, Yingxia Hou, Xue Li, Ran Zhao, Xiaoquan Mu, Yifan Liu, Chengcheng Huang, Frank Fu, Fengxia Yang

**Affiliations:** 1 China-UK Agro-Environmental Pollution Prevention and Control Joint Research Centre, Agro-Environmental Protection Institute, Ministry of Agriculture and Rural Affairs, No. 31 Fukang Road, Nankai, Tianjin 300191, China; a17698731336@outlook.com (J.W.);; 2 Hubei Provincial Department of Agriculture and Rural Affairs, Wuhan 430070, China; 3 Faculty of Social Sciences, The University of Hong Kong, 11/F, The Jockey Club Tower, Pokfulam, Hong Kong 999077, China

**Keywords:** modified biochar, sulfonamide antibiotics, adsorption mechanisms, degradation pathways

## Abstract

Sulfonamide antibiotics (SAs) are ubiquitous and persistent organic contaminants in aquatic and soil ecosystems due to their extensive application and high structural stability, causing rising environmental hazards. Conventional treatment approaches, generally based on physical adsorption or biological processes, remain limited in achieving efficient and stable removal as well as deep molecular modification of SAs. In recent years, modified biochar has developed as a flexible environmental functional material incorporating adsorption and reaction regulation capabilities, owing to its customizable pore structure, surface chemistry, and electronic characteristics. This study comprehensively highlights current achievements in the adsorption and degradation of sulfonamide antibiotics by modified biochar, with specific emphasis on modification techniques, structural modulation, structure–performance connections, and interfacial reaction processes. Through physical activation, heteroatom doping, defect engineering, and metal integration, biochar has developed from a traditional adsorbent into a carbon-based interfacial reactor capable of pollutant adsorption, molecular activation, and directed transformation. Surface-confined reaction interfaces, where π–π interactions, hydrogen bonding, electrostatic interactions, and metal coordination cooperatively control adsorption and transformation processes, are primarily responsible for the elimination of SAs. Moreover, the dual functions of modified biochar in driving both radical and non-radical pathways are explored, showing the vital importance of interfacial electronic structure modulation and electron-transfer mechanisms in influencing reaction efficiency and selectivity. The impact of sulfonamide molecular configurations, ambient circumstances, and concomitant chemicals on removal performance are also explored. Unlike previous reviews that mainly summarize adsorption efficiency or oxidant activation systems separately, this work integrates structural modulation, interfacial electronic regulation, and bond-selective transformation mechanisms into a unified structure–chemistry–reactivity framework. By correlating sulfonamide molecular configuration with biochar electronic structure, this review provides a mechanistic roadmap for the rational design of next-generation catalytic biochar systems. Finally, key challenges related to structural controllability, long-term stability, and engineering scalability are identified, and future research directions are proposed to support the rational design of high-performance biochar materials and the practical control of sulfonamide antibiotic pollution.

## 1. Introduction

**Figure 1:**
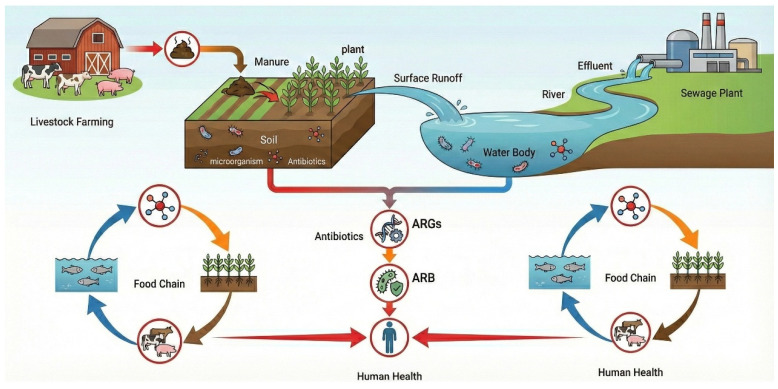
Schematic diagram of the sources–environmental fate–ecological and health risks of Sas. ARB denotes antibiotic-resistant bacteria, and ARGs denote antibiotic resistance genes. Different colored arrows indicate different transport and transformation pathways in the environment: red arrows represent ARG dissemination pathways, blue arrows indicate water-mediated transport, orange arrows represent antibiotic transfer, and brown arrows denote agricultural recycling processes.

As one of the earliest synthetic antibacterial drugs to achieve large-scale applications, sulfonamide antibiotics (SAs) are widely used in human medical care, livestock and poultry breeding, aquaculture and other fields because of their broad-spectrum antibacterial properties, low cost and high structural stability. A large number of studies have shown that SAs are difficult to fully metabolize in living organisms. About 30–90% are discharged into the environmental system through feces and wastewater in the form of parent compounds and biologically active metabolites, so that they are continuously detected and accumulated in soil, water and sediments. Long-term low-dose exposure to sulfonamide antibiotics in environmental media can not only directly inhibit microbial activity and disturb microbial community structure and ecological function, but also significantly promote the production and diffusion of antibiotic resistance genes (ARGs) through selective pressure, and can be transmitted through the food chain, which contributes to ecological safety and public health. Potential long-term risks are shown in Figure 1. Although existing sewage treatment technology and soil repair technology (such as traditional biological treatment, coagulation precipitation and conventional adsorption) have achieved certain results in the removal of conventional organic pollutants, the removal of sulfonamide antibiotics with stable structure, strong water solubility and significant drug resistance risk still faces limited efficiency and environmental adaptation [1]. There remain problems such as poor selectivity and proneness to secondary pollution. In complex environmental matrices, sulfonamide antibiotics frequently coexist with natural organic matter, heavy metals, and microorganisms, thereby undermining the operational stability and sustained performance of conventional treatment technologies [2]. Therefore, the development of sulfonamide antibiotic pollution-control materials and technologies with high efficiency, sustainability and environmental compatibility has emerged as a central principle guiding the development of next-generation environmental functional materials and remediation technologies.

As a carbon-rich porous material formed by the thermal lysis of biomass under oxygen-restricted conditions, biochar has the advantages of a wide range of sources, low cost, adjustable structure and good environmental compatibility, and has broad application prospects in the removal of organic pollutants and heavy metals [3]. However, primitive BC generally has defects such as limited specific surface area, insufficient active sites and weak interface reaction ability, which limit its ability to achieve efficient removal and thorough transformation of sulfonamide antibiotics [4]. In recent years, BC has been directionally modified through strategies such as physical activation, chemical modification, biological functionalization and multiphase compounding, which can achieve precise regulation at multiple levels, such as pore structure, surface functional groups and electronic structures, thereby substantially strengthening its adsorption capability and interfacial catalytic reactivity toward sulfonamide antibiotics. More importantly, some modified BC has changed from a traditional adsorbent to an adsorption–catalytic synergistic material with the ability to enrich pollutants and become activated, which can achieve efficient degradation and risk reduction in sulfonamide antibiotics through the synergy of free radical and non-free radical pathways. At present, although research on modified BC in the removal of sulfonamide antibiotics continues to emerge, there are still a number of key scientific problems to be systematically resolved: (i) how different modification strategies can coordinate to regulate the microstructure and electronic properties of BC. (ii) How structural variations among sulfonamide antibiotics influence their adsorption behavior and transformation pathways at the modified BC interface. In complex environmental systems, the long-term stability, reaction mechanisms, and potential ecological risks of modified BC remain insufficiently understood. To a certain extent, these problems have restricted the transformation of modified BC from laboratory research to engineering applications.

To date, most existing reviews have focused on either adsorption performance or advanced oxidation systems separately, lacking a mechanistic integration between molecular structure of sulfonamides and electronic modulation of modified biochar. Moreover, bond-selective degradation pathways and the transition from radical-dominated oxidation to interfacial electron-transfer mechanisms remain insufficiently summarized. Therefore, this review aims to bridge adsorption thermodynamics, catalytic activation, and molecular transformation pathways within a unified mechanistic framework. Based on this, this paper systematically reviews the latest research progress on modified BC in the field of adsorption and degradation of sulfonamide antibiotics in recent years, focusing on the aspects of modified strategies, structural regulation, adsorption and catalytic mechanisms, and environmental application performance, and combines the molecular structure characteristics of different sulfonamide antibiotics to be discussed in depth. We discuss its interface mechanism and removal laws. On this basis, the key challenges faced by modified BC in practical applications are further analyzed, and its development directions in the coordinated control of multi-pollutants and sustainable environmental restoration are further analyzed in order to provide theoretical references for the rational design of high-performance BC materials and the optimization of sulfonamide antibiotic pollution-control technology.

To ensure a comprehensive and structured overview of recent advances in sulfonamide antibiotic (SAs) removal by modified biochar, a systematic literature survey was conducted. Relevant publications were retrieved from Web of Science Core Collection, Scopus, and ScienceDirect databases, covering the period from January 2010 to March 2026. The search was performed using combinations of keywords including “sulfonamide antibiotics”, “modified biochar”, “biochar adsorption”, “persulfate activation”, “peroxymonosulfate (PMS)”, “advanced oxidation process (AOP)”, “Fe–Nx”, “radical pathway”, and “non-radical pathway”. Only studies directly focusing on sulfonamide antibiotics and biochar-based systems were included. Publications addressing unrelated pharmaceutical compounds or non-relevant contaminants were excluded unless mechanistically informative. After screening and evaluation, representative studies were selected to construct a focused and up-to-date review framework.

## 2. The Fundamental Characteristics and Advantages of Modified BC

### 2.1. BC and Its Modification Methods

BC is a porous, carbon-rich material produced via the pyrolysis of biomass under oxygen-limited conditions. Its environmental function is mostly determined by its surface chemical characteristics and microstructure [5]. Typical biochar generally has a hierarchical pore structure, which is composed of micropores, mesopores and macropores [6]. Micropores principally contribute to the specific surface area and determine the maximum adsorption capacity of pollutants, while mesopores and macropores provide mass transfer routes for the molecular diffusion, interface contact and reaction kinetics of pollutants [7]. The hierarchical pore structure provides a physical basis for the adsorption and subsequent interfacial reactions of sulfonamide antibiotics within the pores, which enables biochar to simultaneously adsorb contaminants with diverse molecular sizes and physicochemical properties [8]. At the carbon skeleton level, BC is composed of aromatic carbon sheets and graphite-like domains, with a certain degree of a π conjugation system and structural defects [9]. The aromatic carbon network not only gives the material high structural stability, but also provides an important interaction basis for aromatic organic pollutants [10]. For sulfonamide antibiotics that generally contain benzene rings and heterocyclic structures, the aromatic domain on the surface of BC can enhance the intermolecular affinity through π–π stacking interactions and hydrophobic action, thus improving its adsorption stability and interface adsorption ability [11]. In addition to the carbon skeleton structure, the surface of BC is usually rich in oxygen-containing functional groups such as hydroxyls, carboxyl groups and carbonyl groups, which constitute the core source of their chemical reaction activity. On the one hand, polar functional groups can interact with groups such as –NH_2_ and –SO_2_NH– in sulfonamide antibiotic molecules through hydrogen bonding, electrostatic interaction and surface complexation to enhance their adsorption capacity [12]; on the other hand, these functional groups and structural defect sites can also participate in electron transfer under certain conditions [13]. The process provides a reaction basis for the subsequent redox reaction and catalytic activation process.

Biochar physicochemical properties are strongly dependent on the nature of the precursor feedstock. Based on biomass origin, biochar precursors can be broadly classified into three major categories: (i) lignocellulosic biomass (e.g., wood, crop straw), (ii) protein- and lipid-rich biomass (e.g., manure, sludge), and (iii) industrial or mixed organic residues. Each category generates biochar with distinct structural characteristics, mineral composition, ash content, surface functional groups, and surface charge properties, which collectively govern sulfonamide adsorption and transformation behavior.

The pyrolysis temperature of BC is a key parameter that regulates the removal mechanism of sulfonamide antibiotics [14]. It determines the adsorption, transformation and environmental risk evolution characteristics of antibiotics through systematic changes in pore structure, surface chemical properties and interface reaction path. Low-temperature pyrolytic BC (≤400 °C) is usually rich in oxygen-containing functional groups such as hydroxyl groups and carboxyl groups [15]. The aromatic structure development is limited, yet it has great enhanced electron transfer capacity and surface reactivity. The elimination of sulfonamide antibiotics by this sort of BC depends more on the interface chemical action and biological regulatory process [16]. Due to the restricted specific surface area and microporous structure, the capacity of low-temperature BC to fix sulfonamide antibiotics is rather weak, and there is a considerable risk of desorption [17]. In contrast, high-temperature pyrolytic BC (≥500–700 °C) shows a highly graphitized aromatic carbon skeleton and a developed microporous structure [18]. Its specific surface area and pore volume have increased significantly, making it have stronger physical adsorption and spatial domain-limiting ability for sulfonamide antibiotics, which can effectively reduce the bioavailability of pollutants and inhibit their environmental migration [19].

**Figure 2:**
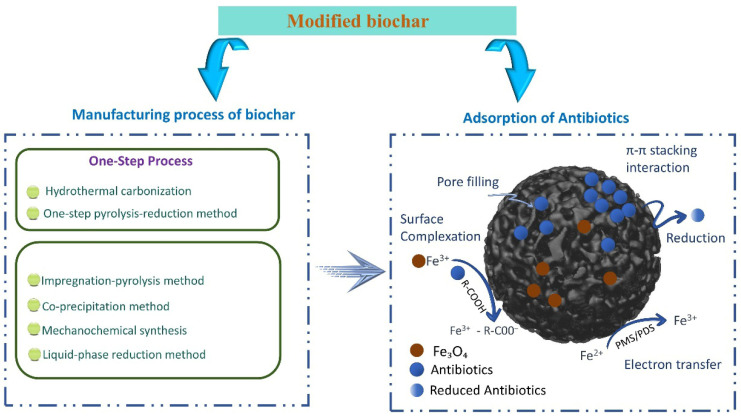
Schematic diagram of modified BC preparation and antibiotic adsorption mechanism. Different arrows represent the process flow and interaction pathways between modified biochar and antibiotics.

However, primitive BC still has certain limitations in the removal of sulfonamide antibiotics, such as insufficient development of specific surface area and microporous structure, limited surface-active site density and weak interface reaction ability. Its removal process is often based on physical adsorption, and it is difficult to realize sulfonamide antibiotics in complex environmental systems and to enable interfacial adsorption followed by electron-transfer-driven oxidative transformation of sulfonamide antibiotics. In order to overcome the above shortcomings, researchers modified BC in a directional way through a variety of ways such as physics, chemistry and biology to regulate its pore structure, surface function group composition and electronic properties, so as to significantly improve the adsorption performance, structural stability and catalytic efficiency [20]. Among these modification approaches, activation (including both physical and chemical activation) represents one of the most fundamental and widely adopted strategies for enhancing biochar performance. Physical activation, typically conducted under CO_2_ or steam at elevated temperatures, primarily develops pore structure through selective carbon gasification. Chemical activation, involving agents such as KOH, NaOH, H_3_PO_4_, or ZnCl_2_, promotes dehydration, bond cleavage, and micropore generation during thermal treatment. Unlike simple mechanical treatments or surface functionalization, activation fundamentally reconstructs the carbon framework, significantly increasing accessible surface area and exposing reactive sites. Therefore, activation should be distinguished as a primary structural regulation pathway that governs subsequent adsorption and catalytic behavior of sulfonamides [21]. Different modification methods and their corresponding structural regulation characteristics and performance changes have been systematically summarized in a large number of studies. The modification methods of BC are shown in Table 1. Based on these modification paths, in order to further improve the removal efficiency of BC on sulfonamide antibiotics, functional BC systems have been widely constructed in recent years, of which iron-based BC is the most representative, and its preparation generally follows the technical route of “pro-mixing-pyrolysis carbonization-post-treatment and active component introduction” [22] (Figure 2). Common methods include the impregnation-pyrolysis method (pyrolysis to form FeO_X_/Fe_3_O_4_ or dispersed iron site after iron salt load) [23], co-precipitation/in situ mineralization method (aqueous phase construction of iron oxide-carbon composite structure), hydrothermal-pyrolysis synergy method (first constructing rich functional group hydrothermal carbon and then loading iron species), mechanical chemical sphere grinding method (promoting the uniform dispersion of iron sources and introducing defect sites), and iron–nitrogen co-doping strategy (introducing nitrogen-containing precursors to construct Fe–Nx coordination structures) [24]. In addition, the development of pore structure, evolution of surface functional groups, formation of defects, and modulation of electronic structure all work together to enhance the adsorption capacity and catalytic activity of modified biochar for sulfonamides. These methods realize the integrated function construction of “adsorption interfacial catalytic transformation” by jointly regulating the morphology, pore structure and surface chemical properties of iron species, laying the material foundation for the application of modified BC in the adsorption and degradation of sulfonamide antibiotics.

A growing body of evidence indicates that biochar modification induces quantifiable changes in pore structure and surface chemistry, which can be directly translated into improved sulfonamide removal. Although alkaline treatment increased the specific surface area from 3.996 to 8.957 m^2^/g and further to 12.740 m^2^/g, the absolute magnitude of this increase remains relatively modest. Considering that BET measurements may exhibit experimental variability depending on degassing conditions and sample heterogeneity, such differences should be interpreted with caution. In fact, many types of high-performance biochar reported in the literature typically possess surface areas ranging from 200 to 1000 m^2^/g. Therefore, in this case, the observed enhancement in sulfonamide degradation cannot be solely attributed to surface area expansion. Instead, accumulating evidence suggests that catalytic improvement in low-surface-area systems is more strongly associated with changes in surface chemistry and active-site composition (e.g., C=O adsorption, defect formation, and electronic modulation) rather than simple increases in pore volume. This highlights the importance of distinguishing between quantitative surface area expansion and qualitative active-site regulation when evaluating structure–performance relationships in modified biochar systems [25]. Related studies have confirmed that sludge biochar pyrolyzed at 600 °C, after being ball-milled (500 rpm, 60 min) and hydrothermally activated with phosphoric acid, can efficiently remove sulfamethoxazole at environmental concentrations through multiple mechanisms such as pore filling, π–π conjugation, hydrogen bonding, and P-O complexation. Its maximum adsorption capacity reaches 4.61 × 10^4^ μg/g, which is 6.30 times that of the original biochar. Moreover, it has good NaOH regeneration performance and low phosphorus leaching risk [26]. Similarly, metal-doping strategies significantly regulate interfacial redox activity. In a magnetic biochar system derived from cow dung, Fe and Cu co-doping (optimal Fe/Cu ratio = 1:1) enhanced PMS activation efficiency, increasing the SMX degradation rate constant from 0.0161 min^−1^ (pristine biochar) to 0.0403 min^−1^ in the Fe–Cu co-doped system. The removal efficiency reached 91.6% under optimized conditions (0.05 g/L catalyst, 1.2 mM PMS) [27]. EPR and probe experiments confirmed the simultaneous generation of SO_4_•^−^, •OH, O_2_•^−^, and ^1^O_2_, with ^1^O_2_ being the dominant reactive species. The enhanced catalytic activity was associated with improved electron transfer between Fe and Cu sites and increased steady-state concentrations of reactive oxygen species.

Collectively, these quantitative comparisons demonstrate that modification strategies not only increase specific surface area and optimize pore architecture but also regulate active-site composition and interfacial electron-transfer pathways. The enhancement of sulfonamide removal is therefore governed by a coupled structure–chemistry–reactivity framework rather than simple surface adsorption alone.

**Table 1:** BC modification methods and their performance changes.

Modification Methods	Specific Methods	Performance Changes	Literature Sources
Physical modification	Ball milling	The particles are smaller and the specific surface area is larger. The crystal structure is altered to enhance stability.	[28,29,30]
	Microwave treatment	The number of functional groups, specific surface area, and stability are significantly increased, and the adsorption capacity is enhanced.	[31,32]
	Thermal treatment	Increase aromaticity and degree of carbonization, reduce polar functional groups, and enhance the adsorption capacity for hydrophobic pollutants.	[33,34]
Chemical modification	Acid modification	Alter the quantity and types of functional groups such as hydroxyl groups on the BC surface. Meanwhile, enhance the BC’s adsorption capacity for organic pollutants.	[35,36,37]
	Alkali modification	Strong alkali can increase the hydrophilicity of the BC surface, while also increasing the surface alkaline functional groups, giving its surface a negative charge to adsorb more cationic pollutants.	[36,38,39,40]
Biological modification	Microbial action	Microbial secretions cover the surface, increasing oxygen-containing functional groups, but may clog pores and reduce specific surface area.	[41,42]
Nano-treatment	Introducing nano-metal oxides	Improve the porous structure of BC and characteristics such as specific surface area, functional groups, and thermal stability.	[43,44,45]

### 2.2. Progress in the Application Research of Modified BC to Sulfonamide Antibiotics

With the deepening of the understanding of the environmental behavior and removal mechanism of sulfonamide antibiotics, modified BC has become an important functional material for the control of sulfonamide pollutants because of its adjustable pore structure, surface chemical properties and reactive activity [46]. A large number of studies have shown that compared with primitive BC, modified BC constructed through strategies such as physical activation, chemical modification, heteroatomic doping and metal loading has shown significant advantages in adsorption, interface reaction and conversion efficiency of sulfonamide antibiotics. At the adsorption level, modified BC usually has a higher specific surface area and a more reasonable aperture distribution, which can effectively enhance the mass transfer and interface contact of sulfonamide antibiotics [47]. Relevant studies show that modified BCs used for different sulfonamide antibiotics (e.g., SMX, SDZ, SMZ) exhibit superior performance to traditional BC in terms of adsorption kinetics and isotherm characteristics, and are more resistant to solution pH and coexisting ions [48]. In terms of degradation, modified BC gradually changes from a single adsorption material to an “adsorption-catalytic coordination” functional material [49]. By introducing transition metals (such as Fe, Mn, Cu) or constructing an iron-nitrogen co-doped structure, the surface of BC can form an interface reaction center with redox activity, and the catalytic degradation of sulfonamide antibiotics can be realized with the participation of persulfate, peroxide or dissolved oxygen [50]. Research shows that modified BC not only acts as an adsorption carrier in the process of sulfonamide antibiotics, but also plays a key role by enhancing the biodegradation process [51]. Functionalized materials represented by NaOH modify BC; under optimized dissolved oxygen conditions, the removal process transitions from an initial rapid reduction phase to a stabilized degradation stage, indicating that its main removal mechanism gradually changes from surface adsorption to deep biodegradation [52]. In addition, the structural regulation of modified BC also considerably affects the conversion path and intermediate product evolution process of sulfonamide antibiotics. The migration of SAs in environmental media such as soil and sediment facilitates their dissemination [53]. Their migration behavior is determined by their own physicochemical features (such as adsorption characteristics, water solubility, partition coefficients, etc.) and external environmental variables (such as climate, precipitation, etc.). Compared with primitive BC, which is mainly based on physical adsorption, functionalized BC is more conducive to inducing the activation and fracture of key functional groups in sulfonamide molecules and promoting their transformation into small-molecule products, thereby reducing environmental persistence and residual risk [54].

Modified biochar systems have been evaluated under realistic water matrices, where Fe/N- or Fe/Cu-doped biochar activated PMS to achieve >90% sulfonamide removal within 20 min under optimized catalyst dosages (30–100 mg L^−1^) and PMS concentrations (1–2 mM), exhibiting broad pH adaptability (3–11) and degradation rate constants up to 0.37 min^−1^. However, matrix components such as humic substances and competing anions significantly affected reaction kinetics, and although metal leaching remained below regulatory limits and partial reusability was demonstrated, limited mineralization and long-term stability under continuous-flow conditions remain critical challenges for practical application [55]. In realistic soil–water systems, Fe/Mn-modified biochar markedly improved SMX removal, achieving up to 98% degradation within 48 h at 1 mg·L^−1^ and significantly shortening the environmental half-life, while increasing specific surface area (5.26 to 91.70 m^2^·g^−1^) and pore volume (0.018 to 0.20 cm^3^·g^−1^), thereby enhancing pollutant adsorption and interfacial reactivity. Quantitative analysis demonstrated that degradation (79.5–83.8%) rather than simple adsorption dominated removal, with •OH-mediated oxidation confirmed in complex matrices, underscoring the practical relevance of modified biochar for in situ antibiotic mitigation [56]. In summary, modified BC shows the significant advantage of transforming from adsorption materials to functionalized adsorption–degradation synergistic materials in the treatment of sulfonamide antibiotics, which provides important support for its application in complex environmental systems.

## 3. Removal Mechanisms of SAs with Different Structures

### 3.1. The Chemical Structure of SAs

**Figure 3:**
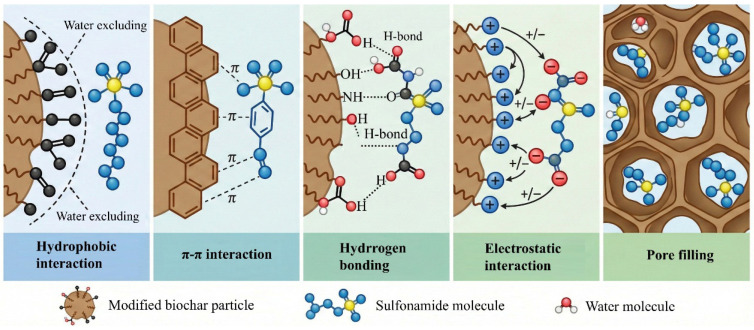
Schematic illustration of the electrostatic interactions between sulfonamide antibiotics (SAs) and BC surface. The surface charge of BC is dependent on pH and surface functionalization. Under acidic conditions (pH < pHPZC) or after cationic modification, the BC surface may exhibit a positive charge, facilitating electrostatic attraction toward anionic SA species.

SAs are a class of important antibacterial drugs. The core structure of SAs is an SA group (-SO_2_NH_2_), which consists of a sulfonyl group (-SO_2_-) and an amino group (-NH_2_). This structure imparts polarity to the molecule, affects their solubility and interaction with biological targets, and inhibits protein synthesis by binding to bacterial cell ribosomes. At the same time, the properties and characteristics of the drug can be significantly changed by introducing different substituents (such as alkyl substituents, aromatic cyclic substituents, heterocyclic substituents, and halogen substituents) on SA groups (-SO_2_NH_2_) (Table 2 and Figure 3).

Sulfonamides are amphoteric compounds possessing two dissociation constants (pKa_1_ and pKa_2_), corresponding to the protonation of the aniline amino group and deprotonation of the sulfonamide moiety, respectively. For representative sulfonamides, such as sulfamethoxazole (SMX), the reported values are pKa_1_ ≈ 1.6 and pKa_2_ ≈ 5.7; for sulfadiazine (SDZ), pKa_1_ ≈ 2.0 and pKa_2_ ≈ 6.5; and for sulfamethazine (SMZ), pKa_1_ ≈ 2.3 and pKa_2_ ≈ 7.4 [57]. At pH < pKa_1_, sulfonamides predominantly exist in cationic form; between pKa_1_ and pKa_2_, the neutral species dominates, and at pH > pKa_2_, deprotonated anionic species prevail. Therefore, the charge-dependent interaction with biochar is highly sensitive to solution pH relative to both the sulfonamide pKa values and the point of zero charge (pHPZC) of the material. When pH < pHPZC, the biochar surface is positively charged, favoring electrostatic attraction toward anionic sulfonamide species (pH > pKa_2_). Conversely, at pH > pHPZC, electrostatic repulsion may occur if both the surface and sulfonamide are negatively charged. Thus, a precise mechanistic interpretation requires correlating specific sulfonamide speciation profiles with measured biochar pHPZC values rather than relying on broad pH ranges.

**Table 2:** SA group substituents and their effects.

Type of Substituent	Common Substituents	Influence	Literature Sources
Alkyl substituents	Methyl (-CH_3_)	Increase lipophilicity and improve absorption and distribution	[58]
Aromatic ring substituents	Phenyl (-C_6_H_5_)	Increase planarity and rigidity, and enhance binding affinity to the target	[59]
Heterocyclic substituents	Pyrimidine ring (Such as SD)	Increase the antibacterial spectrum and activity	[60]
	Isoxazole ring (Such as SMZ)	Enhance the inhibitory effect on specific bacteria	[61]
Halogen substituents	Chlorine (-Cl)	Increase electron density and improve interaction with the target	[62]
Amino substituents	Amino group (-NH_2_)	Increase alkalinity and affect ionization and cell permeability	[63]

### 3.2. The Removal Behavior of Modified BC Towards Sulfonamide Antibiotics with Different Structures

#### 3.2.1. The Impact of Molecular Structural Differences on Interfacial Adsorption Behavior

From a molecular perspective, the interaction mechanism between sulfonamide antibiotics and BC-based materials is mainly determined by their ionization behavior, aromatic structure and functional substituents. Sulfonamide antibiotics typically contain an amino group on a benzene ring and a substituent attached to sulfur. Structural differences are mainly reflected in the position of amino groups, the type of substituents and the spatial arrangement. Different modification strategies result in distinct adsorption mechanisms for structurally diverse SAs.

Relevant studies have shown that the adsorption process of unmodified BC and acid-modified biochar (HBC) on the SAs clodazon, SMZ and SMA is divided into two stages: fast and slow. The adsorption increases with time and reaches equilibrium in about 960 min. Not only are the adsorption mechanisms of BC on SAs of different structures different, but the adsorption mechanisms of SAs in the surrounding environment are also different. In some medium and high latitudes, freeze–thaw events occur frequently. With an increase in the number of freeze–thaw cycles, the adsorption capacity of the soil to SMZ and the SA pyridine (SPY) decreases first and then increases. With an increase in the number of freeze–thaw cycles, the soil clay content, specific surface area, small pores and soluble organic matter (DOM) increased, providing more adsorption sites, finally dominating the adsorption process [64]. In a water environment, the adsorption capacity of carbon nanotubes on SAs is stronger than in a vacuum environment. The adsorption of five SAs on carbon nanotubes is predominantly governed by physical interactions. The most stable adsorption configuration is the parallel adsorption of the benzene ring of the drug molecule on the top of the carbon nanotube. By comparing the adsorption energy, the stability of the adsorption of five SAs on carbon nanotubes is: SMZ > SDZ > SMX > SAs [65]. The adsorption capacity of modified BC for SAs is better than that of traditional BC. The research results of the adsorption capacity of different modified BC types on SAs show that they all show good adsorption properties in the adsorption of SAs (Table 3). The adsorption ability of BC to SAs is affected by many factors, including the surface properties of BC, the preparation conditions and the molecular structure of SAs. Through relevant research, it has been shown that the adsorption capacity of BC for SAs (such as SMZ and SMX) can be significantly improved through modified treatment (such as the addition of phosphogypsum and ball-grinding treatment).

**Table 3:** Adsorption capacity of modified BC for SAs.

Type of BC	Type of SAs	BC Dosage (g·L^−1^)	Initial Antibiotic Concentration (mg·L^−1^)	Equilibrium Reaction Time (h)	Adsorption Capacity (mg·g^−1^)	Literature Sources
Bagasse-modified BC	SMZ	0.1	20	24	3.96	[66]
	Methylpyrimidine	0.1	20	24	2.84	
Phosphogypsum-modified BC	SDZ	0.2	10	24	2.98	[67]
Pyrolysis-modified BC	SDZ	0.1	48	24	261	[68]
Magnetic-modified BC	SMZ	0.01	200	12	414.2	[69]
	SMZ	0.01	200	12	386.3	
Boric acid-modified BC	SMZ	0.5	50	4	92.35	[70]
Ball-milled BC	SMZ	0.01	10	24	36.3	[71]
	Sulfapyridine	0.01	10	24	41.5	
HCl-modified BC	Sulfamethazine	0.5	9.0	24	1.58	[72]

SA compounds with different molecular structures exhibit diverse adsorption behaviors on various adsorbents. Electrostatic interaction is an important mechanism governing the adsorption of sulfonamide antibiotics on BC surfaces. The surface charge of BC is pH-dependent and determined by its pHPZC. When the solution pH is below the pHPZC, surface protonation may render the BC positively charged, whereas at pH values above the pHPZC, deprotonation leads to negatively charged surfaces [73]. Therefore, the electrostatic interaction toward ionizable sulfonamide species is strongly environment-dependent. For SA molecules with higher hydrophobicity, a porous BC matrix with a large specific surface area facilitates their diffusion and retention through hydrophobic interactions and pore-filling effects. In contrast, SA compounds bearing specific functional groups, such as carboxyl moieties or nitro substituents, can interact more strongly with the BC surface via hydrogen bonding or π–π interactions. These structure-dependent adsorption pathways highlight the importance of both adsorbent properties and molecular characteristics in governing SA removal efficiency [66]. The adsorption of SA compounds with diverse structural features onto modified BC is governed by a dynamic equilibrium among multiple interaction mechanisms. By rationally selecting appropriate modification strategies, the surface chemistry and pore characteristics of BC can be tailored to better match the molecular properties of different SA compounds. Such targeted modification not only enhances adsorption efficiency but also improves the stability of SA retention, enabling more effective removal across structurally diverse sulfonamide pollutants [74,75].

#### 3.2.2. Adsorption Kinetics of Sulfonamide Antibiotics on Modified BC

An essential foundation for elucidating the migration, site occupation, and interface interaction mechanism of SAs on the surface of modified BC was provided by an adsorption kinetics study [76]. Due to the aromatic heterocyclic structure and -SO_2_-NH- functional groups of SA molecules, along with their multiple acid–base dissociation constants (pKa_1_, pKa_2_), they can exist in cationic, neutral, or anionic forms depending on pH variations in the surrounding medium, which considerably influences their diffusion behavior and surface binding mechanisms [77]. Current research often fits the adsorption process to differentiate between several rate control steps using an intra-particle diffusion model, a pseudo-first-level dynamic model (PFO), and a pseudo-second-level dynamic model (PSO) [78]. The kinetic model fitting results generally show that the adsorption process of sulfonamide antibiotics is more in line with the pseudo-secondary kinetic model, and the equilibrium adsorption amount calculated by the model is highly consistent with the experimental value, indicating that the adsorption rate is mainly controlled by surface chemistry, rather than simply dominated by solute diffusion [79]. This feature is intimately linked to the interaction of several locations where heterocyclic nitrogen, sulfonamide groups, aromatic rings, and other functional groups in SA molecules are involved. Further intra-particle diffusion analysis usually indicates a multi-stage kinetic process constituted of “rapid surface adsorption—intra-particle diffusion—adsorption equilibrium”. In the initial stage, the boundary layer diffusion resistance is generally modest, but the succeeding stage is limited by the coordination of intra-pore diffusion and surface rearrangement [80]. In general, sulfonamide antibiotics’ rapid kinetic response on porous adsorption materials is due to the synergistic effect of pore structure advantage and strong molecule-interface interaction, which not only ensures that high adsorption capacity can be effectively utilized in a short period of time, but also in rapid adsorption, online pretreatment, and adsorption-catalytic coupling systems. The application provides a vital dynamic foundation.

Considering time scale, the adsorption behavior of SAs on modified BC usually shows the multi-stage characteristics of “rapid adsorption—slow diffusion—balanced rearrangement” [81]. In the initial stage, SA molecules in neutral or weak ionizing forms in the solution can swiftly migrate and occupy the high-energy sites in the outer surface of the BC and medium and large pores, and the adsorption rate is quite fast [82]. Therefore, when the outside surface site is gradually saturated, sulfonamide antibiotics with large molecular sizes (such as SMX and SMZ) are further shrunk, the interior diffusion of the pore structure eventually becomes a limiting step [83]. The final stage is reflected in the process of adsorption–desorption dynamic balancing and the delayed adjustment process of interface setup. The analysis of the intraparticle diffusion model generally shows that the adsorption kinetic curve does not pass the origin, indicating that the adsorption process of SAs is not controlled by a single diffusion mechanism, but is instead the result of the coordinated regulation of external diffusion, intra-pore diffusion and surface chemical action [84].

Different modification procedures have a substantial impact on the adsorption kinetics of sulfonamide antibiotics by modifying the pore structure features, surface kinetic group composition and electrochemical properties of BC. Physical or chemical activation can usually enhance the specific surface area and optimize the proportion of pores, thus minimizing the diffusion constraints caused by spatial resistance of SA molecules and greatly speeding up the initial adsorption rate [85]. However, functional groups in SA molecules, such as –SO_2_–NH–, amino groups, and heterocyclic nitrogen, enhance their interactions with the BC surface.The multi-position interaction on the surface makes the adsorption process more in line with the pseudo-second-order dynamic features [86]. For BC systems modified by metal or metal-N co-colocation structure, SA molecules may also form complexes with the metal center through sulfonamide groups or heterocyclic nitrogen, accompanied by the redistribution of interface electrons, so that the adsorption process shows a higher rate constant in appearance dynamics, and may be associated with the subsequent oxidative degradation process. Form adsorption-reaction coupling behavior [87].

In addition, the regulating effect of environmental variables on the adsorption kinetics of sulfonamide antibiotics is very relevant. The solution pH directly affects the electrostatic action and diffusion behavior by affecting the ionization morphology of SAs and the surface charge state of BC [88]. Under the circumstance of the zero charge point of BC (pHPZC) being approached, the fraction of neutral form SAs increases and the electrostatic rejection effect is lessened, which is more conducive to its migration into the hole and the creation of stable absorption [89]. Attachment, so as to display a faster adsorption rate. In contrast, in solutions with high ionic strength or rich in natural organic matter, competing adsorption and pore blocking effects may impede the efficient diffusion of SA molecules and reduce their surface kinetic rate constant [90]. Reported pseudo-second-order rate constants (k_2_) for antibiotic adsorption on modified biochar generally fall within the 10^−3^–10^−4^ g·mg^−1^·min^−1^ range and vary with molecular size and surface functionality. For example, phosphate-ligand engineered biochar exhibited k_2_ values following the order SAs > ERY > β-Ls > TCs ≈ QNs, indicating that smaller and electrostatically favorable molecules tend to adsorb more rapidly [11]. Thus, comparative evaluation of k values provides clearer insight into structure–kinetics relationships than model fitting alone.

In general, the adsorption kinetics of sulfonamide antibiotics on modified BC is a multi-mechanism collaborative process determined by its molecular structural features, ionization behavior and BC interface qualities. It enhances the rationale of the SA removal method by combining kinetic studies with pore structure, surface chemistry, and molecular structural features. This allows us to solve and build the theoretical framework for the design of the adsorption-catalytic synergy system.

### 3.3. Exploration of Molecular Mechanisms

#### 3.3.1. Surface-Confined Adsorption and Electronic Coupling of Sulfonamides

Modified BC removal of sulfonamide antibiotics is a surface-limited domain transformation process driven by interface microenvironment development, electronic structure management, and molecular bond activation rather than a straightforward physical adsorption or a conventional advanced oxidation approach [91]. Through the incorporation of heteroatomic doping, defect engineering and metal species, BC has evolved from an adsorbent material to a carbon-based interfacial reactor with reaction regulating ability [92]. The degradation of sulfonamide antibiotics first occurs at the boundary reaction interface constructed on the surface of modified BC. Sulfonamide molecules usually contain aromatic skeletons and -SO_2_NH- functional groups, which achieve high-efficiency adsorption and orientation configuration on the surface of modified BC through multiple actions [93]. π–π electron interactions establish a fundamental interfacial coupling framework between modified BC and sulfonamide antibiotics. The modified BC’s defect adsorption zone and highly graphitized aromatic carbon domain have a continuous π conjugate system that may have a notable π-Sulfonamide antibiotic molecules are directionally adsorbed on the carbon-based surface in a quasi-parallel configuration due to the π accumulation effect with the benzene ring and heterocyclic structure [94]. This non-covalent coupling not only enables the efficient adsorption of sulfonamide molecules, but also produces a tight electron coupling channel at the molecular orbital level, which greatly shortens the electron migration distance and stabilizes the interface reaction configuration [95]. Studies have shown that π–π stacking interactions can induce electric load distribution and electron out-domain between carbon-based materials and aromatic molecules, thus promoting interface electron transfer and active species generation, and providing structural and electron prerequisites for the subsequent directional activation of S-N bonds and aromatic skeletons. Consequently, π-π electron action is a crucial chemical mechanism that propels sulfonamide antibiotics from the “solution-free state” to the “interfacial reaction state” in the modified BC system rather than a straightforward physical adsorption force [96]. Oxygen-containing/nitrogen-containing functional groups such as –OH, –COOH and –NH_2_ enriched on the surface of modified BC can form a multi-site hydrogen bond network with the –NH_2_ and –SO_2_– groups in sulfonamide antibiotic molecules, so that sulfonamide molecules can achieve directional anchoring and configuration limitation at the carbon base interface. Molecular kinetics research shows that hydrogen bonds not only help to stabilize the adsorption configuration but also can be dynamically reconstructed during the reaction process and stabilize the reaction intermediate and transition state together, thus significantly reducing the energy barrier of key bond fracture and accelerating the transformation process. At the same time, the matching of the surface charge state of BC under the management of the pH of the solution and the dissolving shape of sulfonamide molecules can further improve electrostatic attraction and interface affinity, and amplify the stability effect of the hydrogen bond network. The above “hydrogen bond-electrostatic coupling” mechanism transforms sulfonamide molecules from the free diffusion state of the solution to the highly restricted interface reaction state, which provides an important structural and thermodynamic basis for subsequent electron transfer and molecular bond activation [97]. The central position anchoring in the metal constructs the most direct interface reaction starting point for sulfonamide antibiotics. Transition metal sites such as iron and manganese introduced into the modified BC, especially the Fe-N_x_ single-atomic coordination structure, can form a stable internal coordinating complexion with the N atoms in sulfonamide antibiotic molecules and the O atoms in the sulfonyl group, so that the sulfonamide molecules change from the physical adsorption state to the chemical anchoring state [98]. The alignment process significantly shortens the spatial distance between the sulfonamide molecule and the metal active center, directly introduces them into the radius of the reaction, and induces the reconstruction of its electron cloud distribution, thus weakening the stability of the S-N bond and aromatic skeleton [99]. Theoretical calculations and in situ spectroscopy results show that the central position of the metal not only improves the selective adsorption ability of sulfonamide at the interface, but also significantly reduces the energy barrier of interfacial electron transfer and oxidation activation, so that sulfonamide molecules give priority to directional activation and initial bond breaking reaction at metal sites. Therefore, metal coordination anchoring constitutes the “first reaction site” and key structural basis for the efficient degradation of sulfonamide antibiotics driven by modified BC [21].

#### 3.3.2. Regulation of Electronic Structure

In the advanced oxidation system in which modified BC participates, the free radical path is still one of the major dominant pathways for the degradation of sulfonamide antibiotics. Take metal-loaded BC as an example; Cu(I)/BC may efficiently activate peroxyacetic acid (PAA) and form a range of highly active oxygen species in situ at the interface, including •OH, alkane free radicals (R–O•), superoxide free radicals (O_2_•^−^) and single-line oxygen (^1^O_2_) [100]. EPR and sudden extinction experiments together show that it is not a single free radical acting in the system, but a multi-free radical parallel oxidation system dominated by •OH and organic free radicals and coordinated by O_2_•^−^ and ^1^O_2_. The above active species can produce strong non-selective oxidative attacks on sulfonamide antibiotic molecules, preferentially targeting sulfonamide bridges (–SO_2_–NH–), aniline structures, and heterocyclic sites, thereby triggering reactions such as C–N bond cleavage, hydroxylation, deamination, and aromatic ring opening. These processes gradually convert macromolecular sulfonamides into smaller intermediates, which are ultimately mineralized into CO_2_ and H_2_O [101]. The free radical drive path emphasizes the core amplification effect of modified BC in the process of “continuous generation of active oxygen—interface adsorption attack—multi-site fracture transformation”, which is the basis of the key mechanism for the current BC-AOP system to achieve efficient degradation of sulfonamide antibiotics [102]. Research shows that BC after loading Fe, Mn and other transition metals not only introduces recyclable metal active centers but also significantly enriches surface persistent free radicals (PFRs), thus constructing multi-source synergistic active oxygen. Systematic [103]. The metal site can trigger the Fenton-like reaction to produce highly reactive hydroxyl radicals (•OH) by continuously activated H_2_O_2_ in situ in the continuous activation environment through the redox cycle of Fe(III)/Fe(II) and Mn(IV/III)/Mn(II) [104]. At the same time, the carbon oxide core PFRs enriched on the BC carbon-based skeleton can operate as an electronic mediator to increase the activation of O_2_ and H_2_O_2_ and directly contribute in the formation of •OH. Furthermore, intermediate free radicals such as •HO_2_− and •O_2_− create an electron-transfer chain in the system to sustain regeneration of the reduced metal center, thereby enabling the continuous production of •OH. The generated •OH preferentially attacks aromatic rings, heterocycles, and key functional groups such as –SO_2_– and –NH– in sulfonamide molecules, inducing hydroxylation, ring opening, and bond cleavage reactions. These processes progressively convert sulfonamide antibiotics into smaller intermediates. Through metal–carbon synergistic activation of the free radical system, modified BC not only functions as a pollutant adsorption carrier but also promotes deep oxidative transformation of sulfonamides, thereby reducing their environmental risk. Through metal-carbon synergistic activation of free radical systems, this method demonstrates that modified BC not only functions as a pollutant adsorption carrier but also achieves interface depth oxidation and reduces the risk of sulfonamide antibiotics [56].

In recent years, the advanced oxidation system has been changing from “free radical-dominated” to “interface electronic regulation”. For modified BC, its introduction of single-atom site, defective carbon and quinone-type structure is not only the active site, but also reshapes the interface electron structure, so that the removal of SAs changes from somatic phase free radical attack to a non-free radical transformation path characterized by surface electron transfer [105]. In this mode, pollutants are directionally activated through interface charge extraction and electron rearranging, and are accompanied by ^1^O_2_ or surface-bound reactive oxygen generation to achieve selective degradation of sulfonamide molecules [106]. More and more research demonstrates that the Fe–N_X_ single atom site is not only the activation point of PMS, but also the major relay node of interface electron transport. Under the regulation of the defective carbon domain and high-coordination environment, the Fe-N_X_ site can significantly enhance the electron supply ability to PMS and induce its non-free radical activation process, that is, weaken the O-O bond of PMS through interface electron transfer and selectively generate ^1^O_2_ or surface-bound reactive oxygen species. In this method, contaminants undergo directional oxidation transformation through surface electron extraction and electric load composition rather than being primarily non-selectively targeted by •OH or SO_4_•^−^. Higher selectivity and anti-matric interference capabilities are demonstrated by this type of non-free radical electron transfer channel, making it particularly appropriate for the effective elimination of electron-rich contaminants like sulfonamide antibiotics in intricate environmental systems [107]. The defect-enriched carbon domain and the surface quinone/phenol structure together form an interface electronic relay system, which can directly induce electron extraction and electric load structure of sulfonamide molecules on the surface of the material. The process does not rely on the generation and diffusion of solid phase free radicals, but instead activates the molecular structure through interface single electron transfer, and further participates in the follow-up reaction with the formation of ^1^O_2_ or surface-bound active oxygen. Since the reaction is mainly controlled by the electronic structure on the surface of the material, the non-free radical path shows high reaction selectivity and anti-matric interference ability, which is considered to be an important mechanism for the directional transformation and stable removal of sulfonamide antibiotics in complex water environments [77].

**Figure 4:**
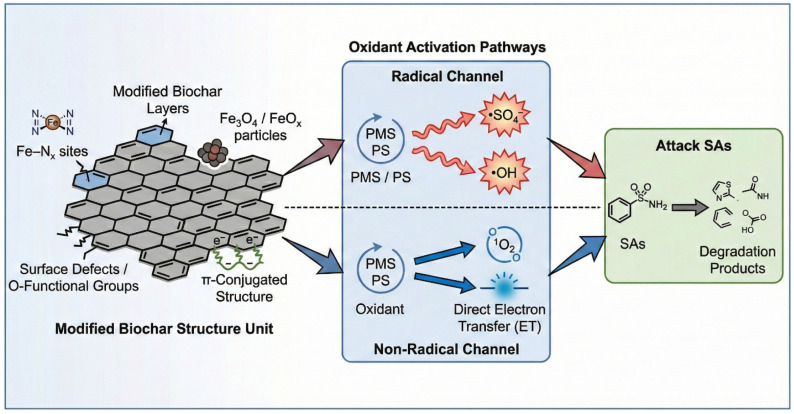
Comprehensive mechanism of SA degradation via modified BC-mediated AOPs4.

Modified BC breakdown of sulfonamide antibiotics can be achieved through both free radical and non-free radical routes at the same time. The free radical pathway is dominated by active species such as •OH, and non-selective strong oxidation of sulfonamide molecules is carried out to promote their rapid bond breakage and deep transformation; the non-free radical pathway relies on Fe-N_X_ single atomic site, defective carbon domain and quinone/phenol structure to build an interface electron transfer channel to induce the occurrence of sulfonamide molecules. Electric charge redistribution and directional electron extraction activate sulfonamide molecules at the catalyst surface, accompanied by the participation of ^1^O_2_ or surface-bound reactive oxygen species. The modified BC has the advantages of high efficiency and selectivity in breaking down sulfonamides in complex environmental systems due to the synergy of the two routes (Figure 4).

According to the aforementioned research, the interface coupling effect between the control of material structure and the molecular properties of pollutants is mostly responsible for the variation in modified BC’s performance in the sulfonamide antibiotic process. From the influence of pore structure and surface chemical properties on adsorption behavior to the regulation of the degradation path of the active site and electron transfer process, relevant research gradually reveals the inherent logic of sulfonamide antibiotics from interface adsorption to reaction transformation evolution in modified BC systems. These mechanical understandings lay the groundwork to further condense the main scientific issues surrounding modified BC in the treatment of sulfonamide antibiotics, in addition to offering a theoretical foundation for explaining the variations in various modification strategies and removal behaviors under environmental conditions.

#### 3.3.3. Structural Transformation Pathways of Sulfonamide Antibiotics Induced by Metal-Modified BC

In metal-modified BC systems, the incorporation of transition metal cations (e.g., Fe, Mn, Cu) or their oxides significantly alters the degradation pathway of sulfonamide antibiotics. Metal active sites can facilitate the activation of oxidants such as PMS, peroxydisulfate (PDS), or dissolved oxygen, leading to the generation of reactive oxygen species (ROS), including •OH, SO_4_•^−^, and ^1^O_2_ [108]. These reactive species preferentially attack electron-rich moieties of sulfonamide molecules, particularly the sulfonamide bridge (–SO_2_–NH–), aniline groups, and heterocyclic rings [109]. The degradation process typically involves hydroxylation, S–N bond cleavage, deamination, and aromatic ring-opening reactions, ultimately leading to the formation of smaller intermediates and partial mineralization. Representative studies have identified characteristic intermediates during SMX degradation, including 3-amino-5-methylisoxazole (3A5MI), hydroxylated SMX derivatives, and sulfanilic-type products formed via isoxazole ring cleavage, S–N bond scission, and subsequent aromatic hydroxylation. Further oxidation processes may generate small phenolic compounds (e.g., polyhydroxylated benzene derivatives) through progressive fragmentation and rearrangement of the sulfonamide structure [110]. Similar transformation behavior has been reported for SDZ and SMZ, where dominant pathways involve S–N bond cleavage, heterocyclic substituent removal, and oxidative modification of the aniline moiety, indicating that sulfonamide degradation generally proceeds through destabilization of the –SO_2_–NH– linkage followed by stepwise aromatic oxidation rather than immediate mineralization [111]. In addition, metal–Nx coordination structures can induce electron-transfer-mediated non-radical pathways, in which the sulfonamide molecule undergoes surface-confined electron extraction and selective oxidative transformation [112]. Therefore, metal incorporation not only enhances adsorption but also fundamentally modifies the decomposition mechanism of sulfonamide antibiotics.

Sulfonamide degradation is governed by bond-selective ROS attack rather than indiscriminate oxidation. Under 222 nm irradiation, •OH and O_2_•^−^ preferentially target electron-rich nitrogen centers and the sulfonamide bridge (–SO_2_–NH–) [113]. In five-membered heterocycles, S–N or O–N cleavage dominates, whereas six-membered derivatives favor SO_2_ extrusion. O_2_•^−^ exhibits higher selectivity toward deprotonated species with stronger electron-donating N^1^-substituents, while •OH drives aromatic hydroxylation and subsequent destabilization. Complementary biodegradation studies reveal that electrophilic monooxygenase-catalyzed ipso-hydroxylation similarly initiates S–N/S–C bond cleavage [114]. Thus, regardless of oxidative or enzymatic conditions, transformation converges on disruption of the –SO_2_–NH– linkage and release of aminated heterocycles. These findings confirm that ROS attack is electronically directed, with N^1^-substituent-controlled charge distribution dictating regioselectivity and degradation kinetics. This integrated perspective highlights that sulfonamide removal is not merely governed by surface area expansion but by electronically directed bond activation at engineered interfacial sites. Such understanding redefines modified biochar from a passive adsorbent to a controllable interfacial reaction platform.

## 4. Conclusions and Future

Modified biochar has evolved from a conventional adsorbent into a tunable carbon-based interfacial reactor capable of coupling pollutant adsorption with bond-selective oxidation of sulfonamides. Through pore engineering, heteroatom doping, and metal–N_X_ coordination design, both radical and non-radical pathways can be regulated to drive structural degradation rather than simple surface accumulation. These advances highlight the potential of modified biochar systems for efficient sulfonamide mitigation in complex environments. However, high removal efficiency alone does not ensure environmental safety or practical viability. Regeneration stability remains insufficiently evaluated, as repeated operation may induce pore blockage, active-site reconstruction, surface oxidation, or metal leaching, potentially reducing long-term performance. Most studies report only short-term batch recycling tests, which are inadequate to assess structural durability under realistic environmental conditions. Moreover, degradation does not necessarily equate to risk elimination. Bond-selective oxidation of the –SO_2_–NH– linkage often produces hydroxylated, desulphonated, or ring-opened intermediates, and incomplete mineralization may result in transformation products with altered toxicity, enhanced mobility, or distinct resistance-selection potential. Parent-compound disappearance frequently exceeds total organic carbon removal, underscoring the need to shift from removal-oriented evaluation toward risk-based assessment integrating molecular identification, mineralization monitoring, ecotoxicity testing, and antibiotic resistance gene response analysis. Equally important, most reported systems remain confined to laboratory-scale batch experiments. In real-world matrices, mass transfer limitations, natural organic matter interference, competing ions, catalyst fouling, and cost-intensive multi-step modification strategies may significantly constrain performance and scalability. Bridging laboratory performance and field applicability requires pilot-scale validation, techno-economic assessment, and life-cycle analysis. Distinct from conventional removal-oriented evaluations, this review emphasizes bond-selective oxidation, interfacial electron-transfer pathways, and molecular-structure-dependent reactivity as the core determinants of sulfonamide transformation. By integrating adsorption thermodynamics, electronic structure modulation, and degradation selectivity, this work provides a mechanistic roadmap for designing next-generation biochar-based catalytic systems.

Future research should therefore prioritize structurally stable, regenerable, and economically realistic systems with verified environmental safety. Only through integrated evaluation of durability, transformation risk, and engineering feasibility can modified biochar transition from a high-efficiency laboratory material to a reliable and sustainable technology for sulfonamide control.

## Data Availability

No new data were created or analyzed in this study. Data sharing is not applicable to this article.
