# Correction: Wang et al. Research Progress and Prospects of Modified Biochar in the Adsorption and Degradation of Sulfonamide Antibiotics. *Antibiotics* 2026, *15*, 268

**DOI:** 10.3390/antibiotics15080803

**Published:** 2026-08-18

**Authors:** Junjie Wang, Yingxia Hou, Xue Li, Ran Zhao, Xiaoquan Mu, Yifan Liu, Chengcheng Huang, Frank Fu, Fengxia Yang

**Affiliations:** 1 China-UK Agro-Environmental Pollution Prevention and Control Joint Research Centre, Agro-Environmental Protection Institute, Ministry of Agriculture and Rural Affairs, No. 31 Fukang Road, Nankai, Tianjin 300191, China; a17698731336@outlook.com (J.W.); 82101221188@caas.cn (X.L.); zhaoran202110@163.com (R.Z.); muxiaoquan@163.com (X.M.); 821012510340@caas.cn (Y.L.); huangcheng0206@foxmail.com (C.H.); 2 Hubei Provincial Department of Agriculture and Rural Affairs, Wuhan 430070, China; houmin800@sina.com; 3 Faculty of Social Sciences, The University of Hong Kong, 11/F, The Jockey Club Tower, Pokfulam, Hong Kong 999077, China

## Error in Graphical Abstract

In the original publication [1], there was a mistake in the Graphical Abstract as published. Specifically, the chemical structure of sulfonamide antibiotics was not accurately represented, and the modified biochar was illustrated only with –OH and –COOH functional groups, which could be misunderstood as representing only oxidized biochar and did not adequately reflect metal loading or heteroatom doping. The corrected Graphical Abstract appears below.

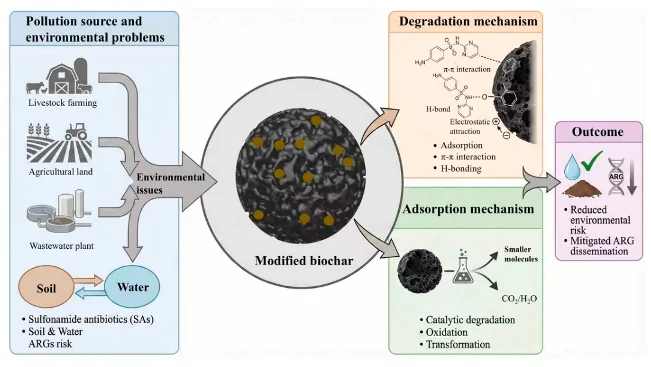



## Error in Figure 3

In the original publication [1], there was an error in Figure 3 as published. Specifically, the chemical structure of the sulfonamide antibiotic (sulfadiazine) and several interaction modes (including hydrogen bonding and electrostatic interactions) were not accurately depicted. The corrected Figure 3 is provided below.

**Figure 3:**
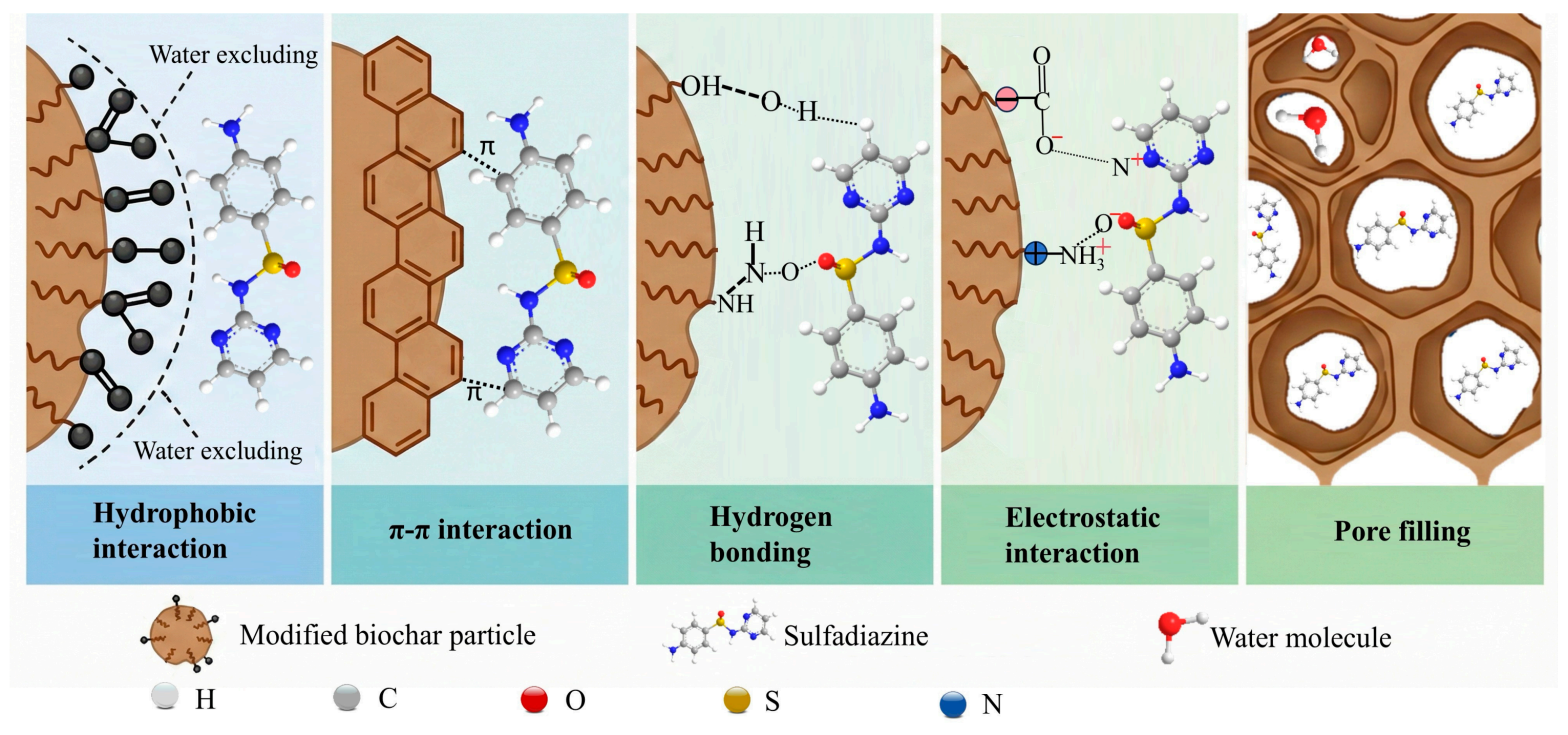
Schematic illustration of the electrostatic interactions between sulfonamide antibiotics (SAs) and BC surface. The surface charge of BC is dependent on pH and surface functionalization. Under acidic conditions (pH < pHPZC) or after cationic modification, the BC surface may exhibit a positive charge, facilitating electrostatic attraction toward anionic SA species.

## Error in Figure 4

In the original publication [1], there was a mistake in Figure 4 as published. Specifically, the chemical structure of sulfonamide antibiotics was inaccurately illustrated, and the degradation products did not correctly represent the transformation pathways of sulfonamide antibiotics during modified biochar-mediated advanced oxidation processes. The corrected Figure 4 appears below.

**Figure 4:**
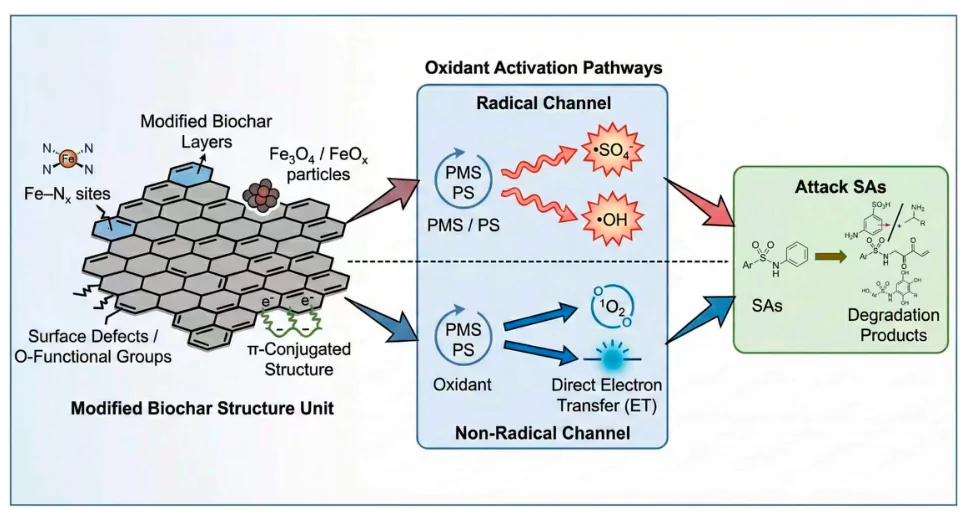
Comprehensive mechanism of SA degradation via modified BC-mediated AOPs4.

The authors state that the scientific conclusions are unaffected. This correction was approved by the Academic Editor. The original publication has also been updated.
